# Commentary: Agroforestry leads to shifts within the gammaproteobacterial microbiome of banana plants cultivated in Central America

**DOI:** 10.3389/fmicb.2016.00656

**Published:** 2016-05-06

**Authors:** Anna Maria Pirttilä

**Affiliations:** Genetics and Physiology, University of OuluOulu, Finland

**Keywords:** core microbiome, clonal propagation, green manure, endophyte, plant health

The microbiomes of organisms are fast becoming thoroughly studied by the development of next-generation sequencing techniques. As hundreds of strains can be identified within hours, the research on microbial community structures and their shifts under varying conditions is a current trend. And rightly so, as microbiomes are recognized as a key factor behind the health of an organism—whether be it human, or plant.

Köberl et al. (2015) studied the gammaproteobacterial microbiome of banana in response to geographical and agricultural factors, and discovered high numbers of *Pseudomonadales, Enterobacteriales, Xanthomonadales*, and *Legionellales*, and an exceptionally high richness of gammaproteobacteria as endophytes in banana. Endophytes, microbes living inside plant tissue without eliciting symptoms (Petrini, 1986), are shown increasingly important for the adaptation and fitness of the host. Members of this microbiome subgroup can protect the host from environmental stresses, induce plant resistance, and promote plant growth (Rosenblueth and Martinez-Romero, 2006; Hardoim et al., 2015). Köberl et al. found the banana gammaproteobacterial microbiome highly stable especially in the endophytic niche. Using profile-clustering network analyses, they however saw differences in the communities between treatments. The greatest differences were observed in the rhizospheric communities between the geographical sites, Nicaragua and Costa Rica. Moderate, but important changes were observed within the epiphytic and endophytic microbiomes between the agroforestry systems, i.e., banana grown with and without green manure, which is used for nitrogen fertilization by legumes growing next to the crop plant. Specifically interesting were the stability of banana microbiome and the changes caused by the neighboring plants in each agroforestry system, which I discuss here.

Our most popular natural treat, banana (*Musa* spp.), is a clonally propagated plant, similar to many important food crops (McKey et al., 2010). Clonal propagation produces plant individuals identical by their genetic heritage and, for example, cultivated banana has almost completely lost the capacity for production of viable seeds. Therefore, banana plants are produced by clonal propagation from suckers, or through micropropagation (Singh et al., 2011). This is especially important when considering endophytic microbiomes of banana, for two reasons.

First, whereas plant seeds can carry members of the microbiome to the next generation, only very few are transmitted through micropropagation (Koskimäki et al., 2010; Quambusch et al., 2014). The meristems that are used for micropropagation often host lower numbers of endophytes than other tissues (Pohjanen et al., 2013), and the diversity is lost by each subculture (Koskimäki et al., 2010). Therefore, an important factor to be considered when studying plant microbiome is the method of propagation, and the fact that the plant loses the majority of microbial members, or at least the diversity of the microbiome becomes very low, during micropropagation (Koskimäki et al., 2010; Quambusch et al., 2014).

Second, the plant microbiome has been shown to depend on the plant genotype (Berg and Smalla, 2009; Lundberg et al., 2012; Turner et al., 2013). Specifically, endophytic microbiomes are similar within species, cultivars, and their ancestors. For example, the endophytic microbiome correlates with host phylogeny, and the bacterial phylotypes are conserved regardless of geographic origin in maize (*Zea mays* L.) (Johnston-Monje and Raizada, 2011). Similar results have been obtained on rice (*Oryza sativa* L.) (Hardoim et al., 2011). Whereas maize and rice are seed-propagated, the genotype-dependency is obvious for clonally-propagated banana (Köberl et al., 2015). Such constant community of microbes is called a core microbiome, being stable through all phases of plant growth (Lundberg et al., 2012).

These two facts considered, Köberl et al. show that the core microbiome is not necessarily carried by seeds, but a plant acquires it from the environment as defined by its genome. This has not been thoroughly assessed in clonally propagated plants before. Our understanding of how the genome-genome based recognition occurs is still in its infancy. It is known that when the plant cell has been intruded, extracellular pattern-recognition receptors (PRRs) in the plasma membrane recognize microbe-associated molecular patterns (MAMPs) (Newman et al., 2013). Signals forwarded by the PRRs activate signaling cascades and initiate MAMP-triggered immunity, the basal plant defense response, defining the further plant defense reactions (Ausubel, 2005). These reactions modify the community structure of the plant microbiome, affecting both existing and entering microbial species (Podolich et al., 2015).

A microbe infecting the plant can shape the microbiome beyond the addition of one species. For example, phytopathogens create shifts in the structures of endophytic microbial communities (Reiter et al., 2002; Lian et al., 2008). The shifts caused by pathogens can have even more pronounced effects on the plant microbiome than genotype (Podolich et al., 2015). Milder, but observable changes in the plant endophyte communities can be detected after inoculation with beneficial microbes (Ardanov et al., 2012, 2016). The study by Köberl et al. reports changes in the gammaproteobacterial microbiome of banana due to neighboring vegetation (green manure). Banana plants growing next to *Erythrina poeppigiana* (Walp.) O. F. Cook, hosted lower numbers of *Erwinia* spp. in the leaves, and the bananas accompanied by *Inga* trees had higher numbers of endophytic bacteria belonging to genera *Pseudomonas* and *Stenotrophomonas* in their pseudostem and root tissues than plants without green manure. This could be the result of infection of banana plants by the microbes originating from the neighboring plants, which definitely deserves our attention.

The green manures have, so far, been considered beneficial in agriculture only due to fixed nitrogen provided by the legume-associated symbiosis. However, the study by Köberl et al. demonstrates the significance of neighboring plants in shaping plant microbiome (Figure 1) and thereby, possibly, affecting plant health. Even if the plant microbiome is mainly defined by genotype, we can manipulate it by potting neighboring plants to attract beneficial species, or through microbial inoculants. For designed manipulation of plant microbiomes, a post-microbiome era awaits to be entered. The traits carried by beneficial species need to be characterized to better understand how each strain induces positive effects on the plant. The sophisticated design of manipulating plant microbiome will help us in creating the future agriculture with reduced use of pesticides and fertilizers.

**Figure 1:**
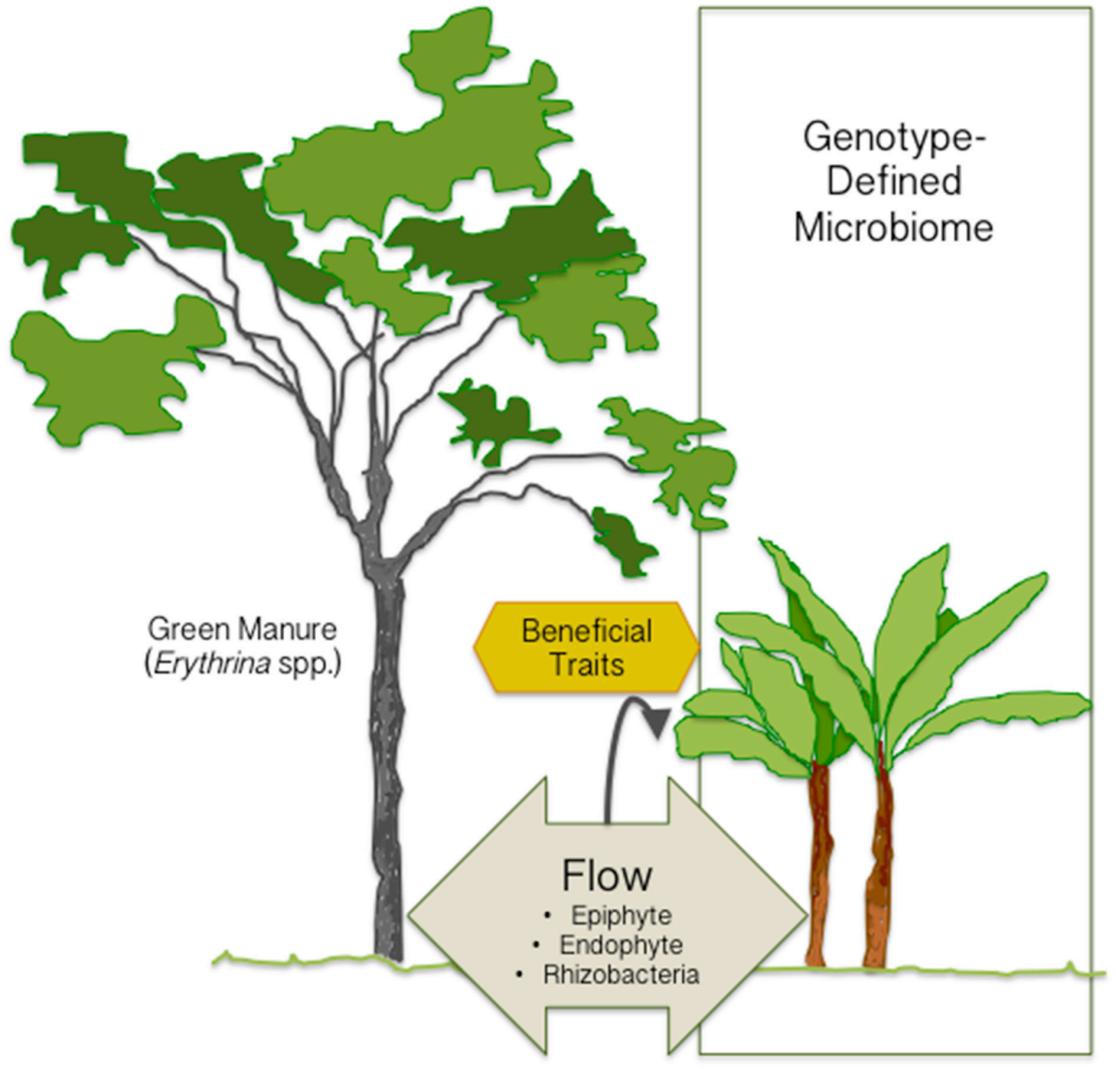
**The plant microbiome is mainly defined by the plant genotype, but it can be manipulated by potting neighboring plants (green manure) to attract beneficial species or by inoculating beneficial microbes**. The traits for improved crop production confer protection against environmental and biotic stresses (heat, drought, herbivory, diseases), and increased plant growth and development.

## Author contributions

The author confirms being the sole contributor of this work and approved it for publication.

### Conflict of interest statement

The author declares that the research was conducted in the absence of any commercial or financial relationships that could be construed as a potential conflict of interest.
